# Three faces of biofilms: a microbial lifestyle, a nascent multicellular organism, and an incubator for diversity

**DOI:** 10.1038/s41522-021-00251-2

**Published:** 2021-11-10

**Authors:** Anahit Penesyan, Ian T. Paulsen, Staffan Kjelleberg, Michael R. Gillings

**Affiliations:** 1 Department of Biological Sciences, Faculty of Science and Engineering, Macquarie University, Sydney, NSW 2109 Australia; 2 ARC Centre of Excellence in Synthetic Biology, Macquarie University, Sydney, NSW 2109 Australia; 3 Department of Molecular Sciences, Faculty of Science and Engineering, Macquarie University, Sydney, NSW 2109 Australia; 4 Singapore Centre for Environmental Life Sciences Engineering, 60 Nanyang Drive, SBS-01N-27, Singapore, 637551 Singapore; 5 School of Biological Sciences, Nanyang Technological University, 60 Nanyang Drive, Singapore, 637551 Singapore; 6 School of Biological, Earth and Environmental Sciences, University of New South Wales, Sydney, NSW 2052 Australia

**Keywords:** Biofilms, Microbial ecology

## Abstract

Biofilms are organised heterogeneous assemblages of microbial cells that are encased within a self-produced matrix. Current estimates suggest that up to 80% of bacterial and archaeal cells reside in biofilms. Since biofilms are the main mode of microbial life, understanding their biology and functions is critical, especially as controlling biofilm growth is essential in industrial, infrastructure and medical contexts. Here we discuss biofilms both as collections of individual cells, and as multicellular biological individuals, and introduce the concept of biofilms as unique incubators of diversity for the microbial world.

## Introduction

According to recent global estimates, 40–80% of all prokaryotes live in biofilms^1^. Biofilms are assemblages of microbial cells attached to each other and/or to a surface, encased within a self-produced matrix. The matrix consists of microbial biopolymers including proteins, exopolysaccharides and extracellular DNA, creating a distinct microenvironment^2^. Thus, for many microbes (including bacteria and archaea, as well as unicellular eukaryotes such as amoeba, flagellates, diatoms and unicellular algae), biofilm formation protects the microbial community from environmental stressors^3–5^. In addition, biofilm formation facilitates interactions between members of the community, as well as resource capture^6^.

Biofilms are ubiquitous and play critical roles in natural and anthropogenic environments. Biofilm communities are important for ecosystem functioning, driving biogeochemical processes, nutrient cycling and bioremediation^1,7^. Beneficial microorganisms form biofilms associated with human, animal and plant hosts as an essential part of the holobiont^8,9^. Conversely, biofilms are also responsible for some 80% of bacterial infections^10^, which are often extremely difficult to treat due to the specific protection mechanisms provided by the biofilm^5^. Similarly, biofilm formation has negative consequences in many industrial settings^11–13^.

Understanding biofilms, the predominant form of microbial life, is important for our ability to control microbial growth. Despite many advances in our understanding of biofilm biology, there are significant gaps in our knowledge of their structure and function. In this article, we present a synoptic view of the multifaceted properties of biofilms. We address our current understanding of biofilms as a unique mode of microbial growth, as well as consider similarities between biofilm assemblages and multicellular organisms. In addition, we present a novel perspective of biofilms as unique incubators of genotypic and phenotypic diversity.

## The nature of biofilms: a mixed paradigm

Debates are ongoing as to whether biofilms should be regarded as a collection of individual cells where each cell represents a separate individual organism, or as multicellular biological individuals where cells are part of an organism rather than individual organisms. Here, we present the key aspects that reflect both sides of the debate, in light of current literature.

### Biofilms as collections of individual cells: the single-cell-centric view

**Fig. 1:**
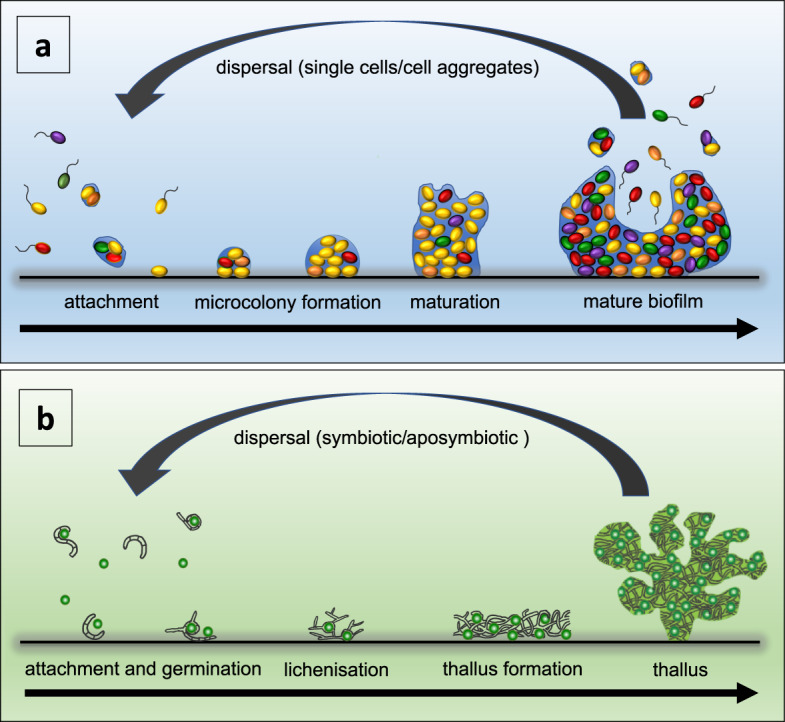
The life cycle of biofilms (panel a), and key stages involved in the vegetative reproduction of lichens (panel b). Panel **a** depicts the main stages of biofilm development, i.e., the attachment of cells/cell aggregates to a substratum, formation of microcolonies and their maturation, followed by dispersal of single motile cells and cell aggregates from biofilms. Panel **b** shows key stages involved in the vegetative reproduction of lichens, including the attachment and growth of symbiotic aggregates (consisting of fungal and algal cells) that are detached from the main lichen thallus, or via aposymbiotic dispersal and germination of fungi followed by re-engagement of algal partners and lichenisation^94^.

Our current understanding of biofilms is largely anchored in the view that biofilms represent a sessile developmental stage in the life of unicellular microbial organisms. Based on this view, biofilm-forming organisms undergo a life cycle that involves both sessile and motile stages. In this life cycle, biofilm formation is initiated by the attachment of cells to a substratum, followed by the proliferation and recruitment of cells from the surrounding environment. Cells in the biofilm then form microcolonies, which mature, and eventually disperse as motile cells and/or cell aggregates released from microcolonies. These spread and serve as inocula for new biofilm initiation and development. As we discuss below, these features are similar to multicellular aggregates, such as lichens, that can also disperse as single cells/cell aggregates (Fig. 1).

Biofilms have long been conventionally considered as collections of individual microbial cells. This single-cell-centric view of biofilms reflects the history of research in microbiology, which has focused on planktonic single bacterial cells traditionally regarded as the primary form of microbial life.

In the last decades, it has become clear that the majority of bacteria and archaea exist as biofilms in their natural habitats^1^. This has raised the possibility of a paradigm shift towards a biofilm-centric view of microbes that regards biofilms as multicellular aggregates that release single bacterial cells as an intermediate dispersal stage, similar to spores or seeds.

### Biofilms have features of multicellular organisms: the biofilm-centric view

Because biofilms consist of multiple cells cooperating to build a differentiated structure, they exhibit characteristics of multicellular organisms. By reflecting on the structure and function of multicellular organisms we may also generate key insights into the biofilm mode of life. For example, cells within a multicellular organism have different specialised functions and different metabolic rates associated with specific developmental profiles^14,15^. Moreover, the transport of materials through such multicellular organisms generates concentration gradients and compartments with different levels of oxygenation and pH. Also, properties of multicellular life include the ability to respond and adapt to environmental stimuli using regulatory mechanisms that control internal behaviours via coordinated responses of interconnected cells^16–19^.

While viewing microbial assemblages such as biofilms as multicellular organisms was proposed by Shapiro^20^, such a model has not been generally adopted, possibly due to the lack of information on system-level commonalities between bacteria and multicellular eukaryotes at that time. Further, the long-held tradition of microbiology research focusing on the use of planktonic cultures has regarded single cells as the main unit of microbial life. Although the concept of biofilm multicellularity has been previously explored^21^, Futo et al.^22^ recently reinitiated discussions in the context of the similarity with eukaryotic models. Using molecular and morphological signatures, these authors showed that *Bacillus subtilis* biofilm growth is highly regulated and organised into discrete ontogenetic stages, analogous to those of eukaryote embryos. Therefore, the authors suggested that biofilm formation in *Bacillus* is a *bona fide* developmental process comparable to organismal development in animals, plants and fungi^22^.

Arguably, biofilms perform collective functions similar to the characteristics of differentiated cells in multicellular individuals^23^. Biofilms are thermodynamically open systems that grow, metabolise and process energy in the form of nutrients. During the biofilm life cycle, the self-produced matrix immobilises cells and creates gradients of nutrients and gases. These gradients lead to the emergence of microenvironments with specific physical and chemical properties, each of which houses cell types functionally adapted to that particular microenvironment. Thus, biofilms are dynamic and organised microbial communities composed of cells that are heterogeneous in space and time^24–26^.

Heterogeneity and the division of labour among specialised cell types are key characteristics of multicellular organisms^27^. In mixed-species biofilm communities, the division of labour is primarily achieved via the interaction of different taxonomic groups, each of which occupies a specific niche within the biofilm based on its physiology and functional profile^28^. For example, in microbial mats living in shallow aquatic environments, microbial species are organised according to their metabolic and energetic properties, whereby the uppermost layers are dominated by aerobic photosynthetic organisms that are exposed to light and oxygen, while the deeper layers are mainly composed of anaerobic microorganisms^29^. In many such cases, the presence of one group of organisms determines the ability of the other group to survive in the biofilm, as illustrated by actively metabolising aerobic organisms consistently consuming the oxygen and making the environment below suitable for anaerobic growth.

Division of labour and phenotypic heterogeneity is not limited to multi-species communities but also occurs within populations of single-species biofilms^30^. Among prominent examples are the biofilms formed by *Myxococcus xanthus*, where cells undergo an elaborate developmental programme that produces at least three different cell types: spores, peripheral rods, and cells that lyse during fruiting body formation^31–33^. Likewise, Haagensen et al.^34^ showed that the mushroom-shaped microcolonies of *Pseudomonas aeruginosa* biofilms are formed via interactions between two distinctly different subpopulations of cells: motile and non-motile. Several phenotypes, or cell types, were observed in *Bacillus subtilis* biofilms, each of which is specialised in a particular trait, for example, motility, surfactin production, matrix production, protease production and sporulation^32,35–37^.

Parts of a biofilm can also have centralised functions similar to circulatory or organ systems in multicellular organisms. Thus, the biofilm matrix can act as an external digestive system by keeping extracellular enzymes in close proximity to the cells, thus enabling cells to metabolise dissolved biopolymers^25^. Concomitantly, internal water channels in biofilms can be involved in the distribution of nutrients and signalling molecules^38–40^ in a fashion reminiscent of the vascular systems or hyphal networks of eukaryotic organisms.

Biofilms, like true multicellular organisms, regulate internal behaviours, for example, via extracellular signalling involved in quorum sensing^41^, similar to pheromones or hormones of higher organisms. Biofilms can also recognise and respond to environmental stimuli. This is evidenced by the identification of several environmental and physiological triggers that induce biofilm dispersal^42–44^. The transition from a sessile, surface-associated or suspended biofilm phenotype to a free-swimming dispersal phenotype for individual cells can be triggered by conditions such as nutrient^43,45^ or oxygen availability^46^, and the presence of nitric oxide^42^. There are strong parallels here with other nascent multicellular organisms, such as slime moulds. For instance, when resources become depleted, the slime mould *Dictyostelium discoideum* switches from a free-living unicellular amoeba to a multicellular form^47^. Starving cells secrete cyclic AMP to recruit additional cells, thus forming aggregates consisting of thousands of cells. The aggregating cells form the mound stage, enclosed within an extracellular matrix of primarily cellulose and mucopolysaccharide^48^. This multicellular aggregate behaves as a complex multicellular organism, where individual cells specialise to perform different functions. The early multicellular stage can develop into a motile slug that can migrate guided by environmental cues, such as heat, light and the availability of oxygen^47,49^.

Biofilms can change and adapt to environmental conditions via transient changes in gene expression, as well as via permanent genomic changes that are fixed in populations under natural selection. As an example of transient adaptive changes, significant remodelling of protein abundance is observed in *Listeria monocytogenes* biofilms as a function of temperature, reflecting adaptation and homoeostasis^50^. As an example of phenotypic alterations fixed by mutations, the rapid evolution of biofilm cells via permanent genomic changes has been observed in response to the presence of subinhibitory concentrations of antibiotics, leading to the generation of genetically and phenotypically diverse mutants that display increased antibiotic resistance and enhanced biofilm formation^51^.

Consequently, biofilms have been proposed to maintain homoeostasis and an organised structure, characterised by reduced entropy. For a single planktonic cell, the cost of maintaining an entropy gradient in a large changing environment can be significant and unpredictable. Single cells within a biofilm contribute to a new internalised environment that maintains homoeostasis. This is achieved by extending the entropy gradient from the cell membrane to the adaptive boundary of the biofilm overall, thus creating an “extended organism”^52,53^. Taking into account the potential for homoeostasis and the resulting reduction in maintenance energy, such a state can be more energetically favourable, thus providing an additional explanation for the well-documented prevalence of biofilms as the dominant microbial mode of growth^54^. In fact, the biofilm dispersal event at the end of the biofilm life cycle can be viewed as an increase in disorder (entropy), characteristic of degradation, similar to death and decay. In other words, the biofilm life cycle can be regarded as a continuous cycle of growth, reproduction, and re-birth from the propagules (single cells or cell aggregates) left from the previous biofilm organism (Fig. 1a).

Both cooperative^55^ and competitive^56^ interactions between cells can exist to ensure biofilm development. Although seemingly counterintuitive to the notion of a biofilm as a unified multicellular entity, evidence suggests that competitive behaviours, such as the production of antimicrobial compounds, may also stimulate biofilm formation^57–60^. Production of antimicrobials is well documented for microorganisms; indeed, many antibiotics are derived from such natural products^61,62^. Cells that produce antimicrobial compounds can protect the overall biofilm community against unwanted external colonisers and predators^63^. Exposure to antimicrobial compounds may also promote the development and fixation of antimicrobial resistance within biofilm cells^51,64,65^, enhancing their survival. Recent evidence shows the abundance of antimicrobial producer and antimicrobial-resistant microorganisms in a 13,000-year old cave ice core^66^, and in permafrost^67^, highlighting these as common microbial traits that have evolved long before the commercial production and use of antibiotics. Hence, as discussed above, not only cooperation, but also competition within a biofilm community can be viewed as an essential part of biofilm biology, and biofilm fitness depends upon the balance of these interactions. Such interactions are also evident in higher organisms, where competition, as well as cooperation, promotes overall fitness. For example, the killing of potentially harmful cells (e.g., cancer cells or foreign agents) by immune cells is integral to the development and training of an organism’s immunity^68^.

Communal interactions also include regulatory mechanisms of the biofilm lifecycle (e.g., to achieve active dispersal), whereby the net result is analogous to the behaviour exhibited by multicellular organisms, including antagonistic interactions. For example, the production of biosurfactants can promote dispersal as well as control unwanted colonisers.

### Biofilms and the definition of an individual: an evolving argument

The definition of what comprises an individual organism has been a subject of debate in both biology and philosophy. Some of this controversy arises as a consequence of the transition from single-cell life forms to life forms that are obligately multicellular for part of their life cycle. This has been identified as one of the major evolutionary transitions occurring over the last 3.85 billion years of life on the planet^69,70^.

The properties of biofilm cells, as outlined above, include heterogeneity in form and function, similar to multicellular organisms. Moreover, biofilms display physical gradients of nutrients and gases as a result of decreased diffusion into the inner layers of the biofilm, as well as physiological differentiation of microbial cells in the different biofilm microenvironments. They also exhibit coordinated behaviour of cells governed by biofilm-specific regulatory mechanisms such as quorum sensing, again, similar to the coordinated behaviour of multicellular organisms^16,23,71^. All these features allow us to draw parallels between biofilms and traditional multicellular organisms. Consequently, biofilms could be considered representatives of a transitionary state, comprising cells that can proliferate and survive as individual entities, but which can also acquire additional protection mechanisms by the association in biofilms, mimicking the properties characteristic of more conventional multicellular life forms.

Biofilms often include multiple species. Although it may seem controversial to consider multispecies biofilms as single living entities, or as individual organisms, it is now accepted that individual multicellular organisms comprise intimate and obligate mutualisms with diverse microbial partners, in assemblages referred to as the ‘holobiont’^9,72^.

The term ‘holobiont’ was introduced to describe a physiologically and evolutionarily integrated unit consisting of the multicellular host organism and its associated unicellular organisms, which are often essential for the survival of the host^73^. For instance, every individual human is a combination of human and microbial cells, and these diverse cells co-evolve, with congruent evolutionary histories^74,75^. Indeed, the individual cells of holobionts need not have the same genetic material, as evidenced by, for example, the diverse species of mutualistic bacteria resident in the human gastrointestinal tract, now understood to be essential for human health and wellbeing^75,76^. Heterogeneity of multicellular organisms, with respect to the origins of their component parts, is further demonstrated by *Riftia* tubeworms. The digestive tract of these organisms, present at early stages, is lost during development, and subsequently replaced by a trophosome that houses environmentally acquired bacterial symbionts. Sulfur oxidising bacteria fix the carbon upon which the *Riftia* tubeworm is reliant, and are consequently an integral and essential part of the organism^77^.

Bourrat and Griffiths^78^ emphasised the need for integrated evolutionary interests between the partners in a holobiont, in addition to functional interconnectivity. They referred to these systems as multispecies individuals, which can comprise multicellular macro-organisms and their associated microbes, as per the classical definition of a holobiont, but, in addition, include associations in which the partners are of similar size^78^. The latter concept can be applicable to multi-species biofilm communities comprised primarily of microorganisms.

According to Godfrey-Smith^77^, part of the transition to multicellularity is the transition in action, from the level of individual cells to the level of the overall organism, at which the micro-acts of cells translate into the macro-acts of a larger organism. Likewise, Michod^69^ has described the transition of a group to an individual organism as the evolution of altruism and the transfer of fitness from the cell to the group level. Production of ‘public goods’ in biofilms serves as an example of such altruistic acts. Traits characteristic of biofilms, such as the production of the extracellular biofilm matrix, toxins, biosurfactants and breakdown of antimicrobial compounds, are beneficial for the overall biofilm, even though they are produced at the expense of individual producer cells. Thus, cells producing public goods display altruistic behaviour in the common interest of the overall biofilm community^79–82^. Such processes can be tightly regulated via quorum sensing mechanisms in biofilms, ensuring the collective and orchestrated action of many producer cells to achieve required outcomes^83^. The micro-actions of individual cells in a biofilm determine the macro-action of the overall biofilm, allowing it to maintain its structure (production of matrix components), migrate (production of surfactants) and defend itself against hostile organisms (via, for example, production of toxins and detoxifying proteins).

While cell differentiation in eukaryotic multicellular organisms is generally irreversible, biofilm cells have traditionally been assumed to reversibly return to their original planktonic state^71^. However, recent advances in high-throughput sequencing have demonstrated that extensive genomic changes can occur in biofilm cells. Accumulation of genomic changes can be rapid, and these generate new phenotypes that have advantages within the biofilm environment^51^. As such, phenotypes that may not be advantageous to planktonic cells can arise within biofilms, thus generating a phenotypically diverse and unique population of biofilm cells. This is reminiscent of the somatic variations that can arise in some eukaryotic cell lines.

### Biofilms: individuals or collections of cells? Ongoing debates

The debate over whether a biofilm community can be considered as an individual in its own right is ongoing, and it remains controversial whether biofilms do exhibit strict multicellularity or not^84,85^. Here, we present some of the key arguments provided in this debate.

Clarke^85^ expressed scepticism about considering biofilms as multicellular individuals by arguing that selection acts on microbial cells rather than whole microbial communities, thus suggesting the lack of biofilm-level heritability. As discussed above, cells in biofilms are not necessarily genetically related and can represent highly diverse communities, with ongoing recruitment of species from the environment as biofilm communities develop^86^. As such, selection can occur differentially on different cells. A similar phenomenon also occurs in the microbial components of holobionts during development, as well as for the somatic differentiation of eukaryotic cells, such as cells of the adaptive immune system.

Some authors have claimed that because of differential selection, biofilm communities may not form lineages^85,87^. In response to such a proposition, Pedroso suggested that biofilm-level inheritance can be achieved, albeit not via the classical closed process where every cell in a new organism originates from the division of the initial zygote^88,89^. Even though not all cell lineages run in tandem across successive biofilm communities, some species of microbes in a biofilm tend to stick together and co-disperse^90,91^ (clumping dispersal), thus inheriting at least a portion of the initial diversity to form new colonies following the dispersal from the biofilm. Moreover, the process of recruitment of cells into biofilms and co-aggregation illustrates that biofilms do not necessarily acquire any cells that happen to be nearby, but that new cells are recruited based on specific interactions^92^. This increases the probability that certain lineages will co-occur across successive multispecies biofilm life cycles, thus maintaining the relative stability of biofilm structure and composition. The outcome is akin to that of the holobiont organisation or multispecies individuals discussed above. A multicellular host cannot guarantee that its progeny will contain the same mutualistic and commensal bacteria that it houses. However, a combination of vertical inheritance and the selection of bacteria from the environment (itself dependent on host-encoded cues and interactions) ensures some continuity in the multispecies biofilm community.

Some authors have suggested that even though biofilms do not produce well-defined parent-offspring relations (e.g., as mammals do), they may still be considered borderline biological individuals (such as lichens) as they enable their component cell lineages to stay together across successive biofilms^89^. For example, there are strong parallels between the biofilm life cycle and the vegetative reproduction of lichens. Lichens are symbiotic organisms that provide useful models to understand the evolution of non-canonical multicellular organisms^93^. They consist of a filamentous fungus, the ‘mycobiont’, and a photosynthetic organism, the ‘photobiont’, a microalga or a cyanobacterium. This partnership is generally considered obligatory for the lichen fungus^94^. The photobiont benefits the fungal partner by producing organic carbon compounds through photosynthesis. In return, the mycobiont benefits the alga or cyanobacterium by providing protection from environmental stresses^95^. For both biofilms and lichens, a new organism can be generated from multispecies cell aggregates that detach from the original multicellular life form. Alternatively, the partnership can be re-established following the attachment and proliferation of one of the member partners^94^. Thus, the recruitment of multispecies cells from the environment during biofilm growth as well as the establishment of new lichen thalli, equally reflect the ways in which multispecies individuals assemble (Fig. 1a, b).

Given that natural biofilms are generally multispecies organisations, it has also been proposed that descriptions of biological individuality that currently focus on single-species eukaryotes are too restrictive and that a pluralistic and open-ended account of evolutionary individuality is needed^84^. The concepts of the holobiont, multispecies individuals, and examples such as that of *Riftia* tubeworms, discussed earlier in this article, further emphasise that need.

## A fresh look: biofilms as diversity incubators for the microbial world

The ongoing debates about the nature of biofilms as aggregates made of single individual cells, or as an assemblage of cells with properties of a multicellular organism, will likely continue. What is not in contention is that biofilms have fundamental functional properties that make them unique and central to microbial evolution and adaptation.

### Biofilms promote the generation of genotypic and phenotypic diversity

The biofilm mode of life promotes the emergence of genotypic and phenotypic variants^51,96–99^. Generation of such diversity carries inherent risks as, inevitably, a proportion of the diverse individuals will not be able to survive in a given environmental condition.

Many organisms are able to reproduce both sexually (via generating seeds, spores) and asexually (parthenogenesis, budding in yeasts and vegetative propagation in plants). Sexual reproduction, which generates genotypic and phenotypic diversity, often occurs in stressful and/or variable environmental conditions, as opposed to asexual reproduction, which is generally typical of stable, favourable environmental conditions^100,101^. By producing offspring that are genetically identical to the parent organism, fitness in the current environment is assured.

As mentioned above, in general, genomic and phenotypic diversity is generated during times when newly reproduced organisms are likely to find altered environmental conditions. This strategy of bet-hedging occurs when the chances of survival of at least some progeny are increased via the generation of progeny with diverse phenotypes^102^. This process maximises the chance of a few offspring surviving in variable environmental conditions, even at the expense of many other offspring that might not be suited to that environment. In such a scenario, the provision of a protective environment, like biofilms, provides a significant advantage by allowing the generation of diversity before it is put through the sieve of natural selection. Thus, biofilms provide a unique environment where newly generated genetic variants can be protected from the external selection while continuing to undergo evolutionary processes.

### The unique role of biofilms as diversity incubators that protect the dynamic process of generating genotypic and phenotypic variants

While the full scope of biofilm protective mechanisms remains poorly understood, it is widely accepted that biofilm formation provides protection of cells from adverse environmental conditions, including changes in physical/chemical parameters such as ultraviolet radiation, extreme temperature, extreme pH, high salinity, and high pressure^103,104^. Biofilms can also serve as a protective shield against antimicrobials by slowing down or preventing the diffusion of some antibiotics into the biofilm interior and via the chelation of charged antibiotics by charged moieties in the biofilm matrix^5,105,106^. In addition, slow growth within sections of the biofilm and the generation of dormant persister cells further increase the survival of the community as many antibiotics targeting growth-related mechanisms have limited effectiveness against slow-growing and dormant cells^5,106^. Thus, biofilms provide a relatively stable and protected microenvironment for the cells within.

**Fig. 2:**
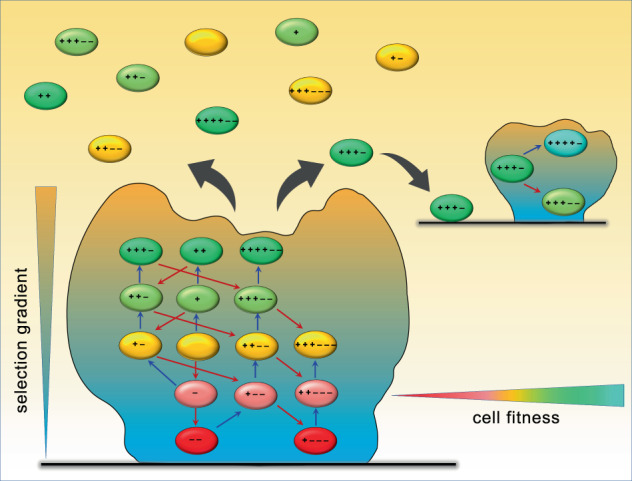
A simplified schematic illustrating the concept of biofilms as diversity incubators, and the dynamic process of diversity generation in biofilms. The biofilm mode of life and external stressors stimulate the generation of mutations in biofilm cells. The selection gradient that forms as a result of external stressors acts differentially on the various layers of cells. This allows the sequential generation of increasingly fit phenotypes under multiple rounds of mutation and increasing selection pressure. Cells with decreased fitness (as a result of initial mutations) can survive within the protective biofilm environment and accumulate mutations that can restore, or even increase their fitness. Upon dispersal from the parent biofilm, cells with decreased fitness perish without the protective shield of the biofilm, whereas cells with increased fitness have the potential to establish new biofilm communities where the further evolution of fitness can occur. Mutations leading to an increase in fitness are indicated by blue arrows followed by a ‘**+**’ sign, mutations leading to decreased fitness are indicated by red arrows and a ‘**−**’ sign. A limited number of variations is presented.

Biofilms are also recognised as the evolutionary hotspots for bacteria. Generation of genomic and phenotypic diversity in biofilms has been well documented^51,96–99^, conforming to a bet-hedging strategy^107^ usually associated with sexually reproducing organisms. However, unlike the genetically diverse seeds, spores, and eggs produced by sexual reproduction, biofilms can protect newly generated genotypic and phenotypic variants, potentially allowing the fixation of further mutations that compensate for any deleterious genetic changes (Fig. 2). These events could be driven by the comparatively soft selection inside the biofilm, such that when novel lineages are released during the biofilm dispersal, they have a better chance of survival.

The shielding characteristics of biofilms create gradients of selection, thus gradually minimising the environmental impact from the surface (maximum impact) toward the biofilm interior (minimum impact). These selection gradients give biofilm cells a unique opportunity to adapt to potentially undesirable conditions by acquiring and accumulating mutations that counteract the impact (Fig. 2). Such advantageous genotypes/phenotypes can subsequently proliferate and outcompete the weaker parental phenotype, thus amplifying the effect of the external selective gradient. All these dynamic processes occur within the protective containment of the biofilm that prevents the environmental impact from having a detrimental effect on biofilm cells, prior to the evolved cells being released during the active process of biofilm dispersal. This process is markedly different from the diversity generation in other organisms where the diversity is fixed (for example, within seeds, spores, and eggs, produced as a result of sexual reproduction), without the possibility of changes and further selection before regeneration. The unparalleled role of biofilms as both evolutionary hotspots and testing grounds for ongoing mutation and selection is under-appreciated but can be central for microbial evolution and adaptation.

This is particularly relevant for antibiotic exposure and the generation of antibiotic-resistant genotypes/phenotypes. It has been documented that the first mutations towards resistance often reduce fitness in the absence of antibiotic selection. Subsequent compensatory mutations restore the fitness to the original wild-type level^108^. Biofilms create a unique protective environment that allows cells with reduced fitness to survive possible disadvantageous mutations and accumulate further mutations that restore fitness, thus travelling through the evolutionary landscape toward developing robust antibiotic-resistant phenotypes. Thus, in biofilms, upon antibiotic exposure, the newly generated genotypes can be exposed to the gradient of antibiotic concentrations, from potential inhibitory concentrations at the surface, subinhibitory in middle layers, to minimal or no antibiotic exposure in the biofilm interior. This selection gradient allows further mutations to occur in multiple rounds of selection, which can restore the fitness that may have been compromised as a result of initial mutations, and lead to superior fitness profiles that are adapted to overcome the antibiotic exposure (Fig. 2). The emergence of multiple co-occurring mutations in genomes of biofilm dispersal cells in the presence of antibiotics, which correlate with increased antibiotic resistance phenotypes in biofilm cells, have recently been demonstrated in different microorganisms^51,64,65^. This supports the view that biofilms act as diversity incubators that both protect the diversity generated and promote the process of diversity generation itself.

In summary, biofilms are a unique microbial lifestyle that exhibits features of multicellular organisms. Biofilms also promote and protect the dynamic process of genotypic and phenotypic diversity generation, thus performing a central role in enhancing microbial adaptation and survival in changing environmental conditions.

## Future perspectives

Biofilms are unique multifaceted life forms that challenge the conventional understanding and concepts of living organisms. Here we present a bird’s eye view of biofilms that encompasses three visions: (1) biofilms as a lifestyle for collections of single individual microbial cells (the single-cell-centric view), (2) biofilms as the main microbial entity with characteristics of a multicellular organism (the biofilm-centric view), and (3) our novel perspective on biofilms as structures that serve as diversity incubators for microbes. Even though the three visions may initially seem to be in conflict with each other, they are not incompatible, but rather complementary. The three visions, each taken from separate perspectives, collectively provide a comprehensive understanding of the biofilm as a unique microbial lifestyle that also carries features of a multicellular organism, as well as performs an important function for microbes by providing a protective environment in which the genotypic and phenotypic diversity is generated before being released.

The conventional understanding of biofilms as a collection of cells that can operate in a coordinated manner benefiting the biofilm community continues to serve us well in revealing biofilm-specific traits such as quorum sensing-regulated behaviour and increased tolerance to environmental stressors. Our expanded multifaceted view of biofilms enables us to explore novel phenomena that can significantly alter our perception of biofilms and illuminate aspects that may have been under-appreciated. For example, profound physiological differences between free-living planktonic microbial cells and those living in biofilms are well documented^51,109–114^. Nevertheless, the importance of unravelling biofilm biology may not be fully appreciated, and the differences between planktonic cultures and biofilms may seem abstract to the wider audience. This can be also due to the microbial world being invisible to the naked eye. One of the visions articulated in this article, the biofilm-centric view, refocuses our view from single cells to biofilms as the main form of microbial life. Biofilms are thus regarded as structures similar to a multicellular organism, where single microbial cells released during biofilm dispersal are viewed as the form of dissemination, akin to seeds or spores in plants. This biofilm-centric view allows us to draw direct parallels with the more familiar macroscopic world to highlight the importance of understanding biofilm biology. Thus, drawing from the biofilm-centric view, focusing exclusively on planktonic cultures to understand the microbial world could be akin to making assumptions about plant biology by only ever looking at seeds or spores. This message fosters a wider appreciation of the importance of studying bacteria in their natural biofilm state.

Moreover, while viewing biofilms as heterogenous multicellular organisms, processes specific to biofilms (e.g., quorum sensing-controlled coordinated cell behaviour, presence of gradients and specific microenvironments inhabited by specialised cells, and the presence of channels for nutrient and gas exchange) provide central targets in controlling microbial growth and persistence. Such strategies have been proposed^115^. For example, furanones produced by the alga *Delisea pulchra*, block bacterial quorum sensing signalling^116^, thus preventing biofilm formation and increasing the susceptibility of bacteria to antimicrobials and other environmental control options. Other anti-biofilm strategies include the inhibition of diguanylate cyclase (DGC) enzymes that synthesise cyclic di-GMP, which is a widely conserved secondary messenger signal for biofilm formation, as well as inhibition of bacterial attachment^117,118^. In addition, strategies can potentially target gas and nutrient exchange by preventing or blocking the transport channels throughout the matrix, subsequently disrupting biofilm microenvironments, heterogeneity and structure.

Lateral gene transfer and short generation times allow microbial evolutionary processes to occur over a relatively short period of time. These processes are often accompanied by the generation of increasingly fit phenotypes, e.g., the evolution of mucoid phenotypes of *Pseudomonas aeruginosa* associated with increased persistence in chronic cystic fibrosis infections^119^. Among the most notable and common consequences of such changes is the acquisition of antibiotic resistance by pathogenic bacteria. Recognising biofilms as incubators that promote the successful generation and spread of microbial diversity, makes biofilm formation a core target for preventing rapid evolution and adaptation of microbial pathogens. Research aimed at preventing biofilm formation may, therefore, pave the way for novel strategies for controlling undesirable microbial evolution and adaptation, including the evolution of antimicrobial resistance, as well as the emergence of highly virulent and persistent phenotypes.

Providing definitions for certain phenomena is an essential part of the scientific process that helps to further develop core concepts, which often become a genesis for new areas of investigation. Nevertheless, research that follows can present evidence that may alter original definitions. Therefore, it is important to revisit definitions introduced in the past, in order to redefine, develop or broaden them as required. Indeed, Flemming et al.^24^ have recently argued that the term ‘biofilm’ is not universally applicable across the broad spectrum of possible organisations for matrix-embedded cell aggregates. Here, we broaden our understanding of biofilms by defining them not only as a lifestyle for individual microbial cells but also recognising biofilm properties that are characteristic of multicellular organisms. In addition, we propose a new fundamental property of biofilms as incubators of genotypic and phenotypic diversity in the microbial world. By combining these three visions we also highlight the multifaceted nature of biofilms, thus expanding our understanding of this dominant microbial life form, as well as moving beyond the simplistic dichotomy of planktonic vs. biofilm cells.
